# Strain echocardiography in a sepsis‐induced cardiomyopathy

**DOI:** 10.1002/ccr3.6502

**Published:** 2022-11-04

**Authors:** Julien Higny, Pierre Bulpa, Yannick Berners

**Affiliations:** ^1^ Cardiovascular Disease CHU UCL Namur Namur Belgium; ^2^ Intensive Care Medicine CHU UCL Namur Namur Belgium

**Keywords:** capnocytophaga, echocardiography, sepsis, speckle‐tracking

## Abstract

Sepsis‐induced cardiomyopathy represents a challenging disorder for critical care practitioners in terms of diagnosis, monitoring and treatment. Strain echocardiography may help to identify ventricular dysfunction at precocious stage in critically ill patients. In this manuscript, we describe early impairment in left ventricular systolic function using speckle‐tracking echocardiography.

## Graphical abstract

Management of septic cardiomyopathy remains challenging in terms of diagnosis, monitoring, and treatment. This report reminds us that the conventional measurement of LVEF is not a sensitive indicator to investigate cardiac function in septic shock. Strain echocardiography enables identification of septic cardiomyopathy by detecting subtle myocardial dysfunction at precocious stage.

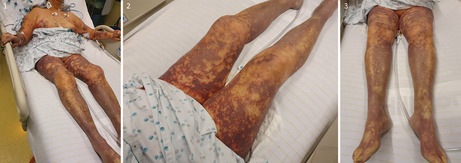

## 1 CASE PRESENTATION

**FIGURES 1–3:**
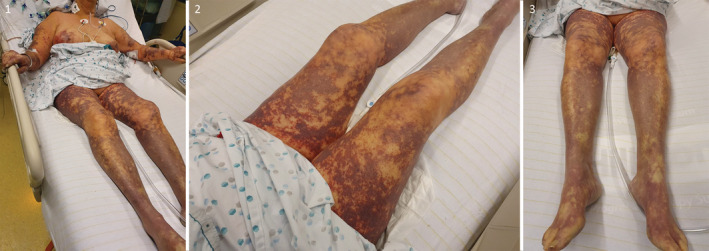
Three consecutive images of the patient illustrating petechiae on the chest and ecchymotic purpura on the upper and lower limbs

**FIGURE 4:**
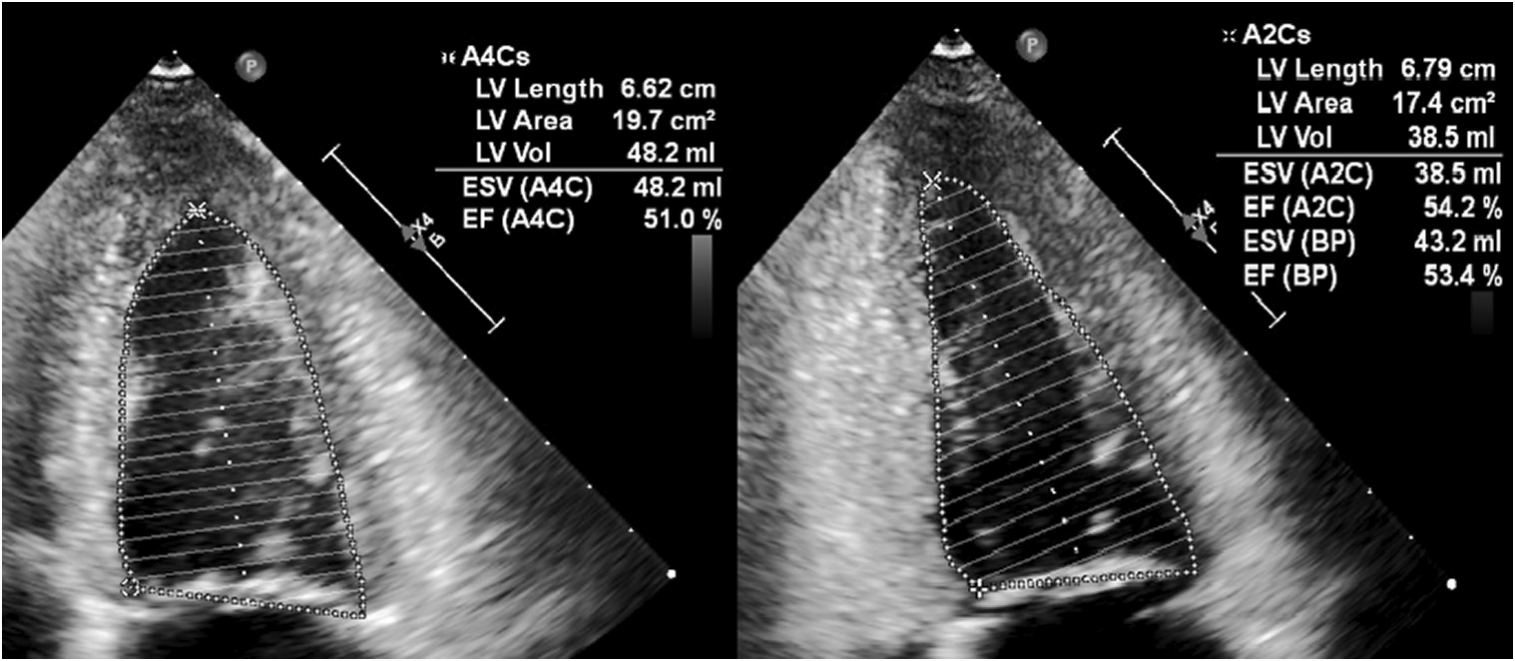
Biplane Simpson's method for calculation of left ventricular ejection fraction (53.4%)

A 68‐year‐old woman was admitted to our emergency department for dyspnea, hypotension, vomiting, and diarrhea. Physical examination revealed purpura and ecchymoses (Figures 1–3). Blood analysis demonstrated an inflammatory syndrome (CRP: 455 mg/L) with neutrophilic leukocytosis (20,081/μl), thrombocytopenia (25,000/μl), acute kidney failure (creatinine: 3.22 mg/dl), impaired coagulation (D‐dimer: >20,000 ng/ml, aPTT: 50 s, TT: 32 s, TP: 44%), and lactic acidosis (pH: 7.00, lactate: 4.8 mmol/L). Lifesaving support included blood culture, ceftriaxone, corticosteroids, fluid resuscitation, vasopressor, and mechanical ventilation. A transthoracic echocardiography was performed to assess left ventricular (LV) function and standard echocardiographic variables. In particular, LV ejection fraction (LVEF) with the biplane Simpson's method (53.4%; Figure 4) and 3D echocardiography (53%) were normal (Video S1). Conversely, speckle‐tracking echocardiography (STE) findings demonstrated abnormal LV systolic function with a global longitudinal strain (GLS) calculated at −13.4% (Video S2). We added dobutamine to norepinephrine because myocardial impairment was evidenced by a depressed GLS value. Within 2 h, we initially observed a decrease in the norepinephrine dose associated with a decline in lactate level. The clinical course subsequently deteriorated with the development of digital necrosis and acute kidney injury requiring bilateral transtibial amputations and continuous veno‐venous hemofiltration. Finally, vasoactive agents were weaned on the tenth day, and the patient was discharged from intensive care 20 days later.

## 2 DISCUSSION

Profound but reversible myocardial depression in septic shock patients has been described in the literature. Transthoracic echocardiography is the first‐line tool to assess patients in shock. Conventional echocardiographic variables may be affected by loading conditions. STE is a novel technological modality allowing early detection of LV dysfunction, prior to decrease in LVEF. Normal value of GLS is reported in the range of −20% in healthy subjects.^1^ The prognostic value of sepsis‐induced myocardial dysfunction detected by STE has been suggested in a meta‐analysis. In this review, worse GLS values directly correlated with higher mortality in comparison with abnormal LVEF.^2^ Clinical implications include monitoring of myocardial dysfunction and institution of appropriate cardioprotective strategies early in the course of the disease. However, the clinical significance of LV systolic dysfunction detected in septic patients is uncertain. Also, the use of inotropic agents and treatment goals are debated.^3^ Therefore, further studies are needed to validate the routine use of STE in the hemodynamic assessment of septic patients.

## 3 CONCLUSION

Identifying cardiac failure in sepsis remains challenging. GLS represents a more sensitive indicator of septic LV dysfunction compared to conventional measurement of LVEF. This clinical picture illustrates that strain echocardiography may help to identify cardiac dysfunction at precocious stage in critically ill patients at risk of myocardial depression during a fulminant sepsis. However, the clinical impact of GLS measurement in septic patients remains unclear.

## AUTHOR CONTRIBUTIONS

All authors have made substantial contribution to the preparation of this manuscript. JH acquired the images, interpreted the data, and drafted the manuscript. YB performed literature research. PB made critical revision and approved the final manuscript.

## CONFLICT OF INTEREST

The authors have no conflicts of interest to declare.

## INFORMED CONSENT

Written informed consent was obtained from the patient to publish this report for educational/research purposes in accordance with the journal's patient consent policy.

## Supporting information


Video S1

Video S2

Appendix S1

## Data Availability

The datasets generated during the current report are available from the corresponding author on reasonable request.

## References

[1] L'Heureux M , Sternberg M , Brath L , Turlington J , Kashiouris MG . Sepsis‐induced cardiomyopathy: a comprehensive review. Curr Cardiol Rep. 2020;22(5):35. 32377972 10.1007/s11886-020-01277-2 PMC7222131

[2] Sanfilippo F , Corredor C , Fletcher N , et al. Left ventricular systolic function evaluated by strain echocardiography and relationship with mortality in patients with severe sepsis or septic shock: a systematic review and meta‐analysis. Crit Care. 2018;22(1):183. 30075792 10.1186/s13054-018-2113-y PMC6091069

[3] Boissier F , Aissaoui N . Septic cardiomyopathy: diagnosis and management. J Intensive Med. 2022;2(1):8‐16. 10.1016/j.jointm.2021.11.004 PMC9923980 36789232

