# Supplementary material for: Strain echocardiography in a sepsis‐induced cardiomyopathy
Source: Clin Case Rep. 2022 Nov 4;10(11):e6502. doi: 10.1002/ccr3.6502 (PMC9634267; doi:10.1002/ccr3.6502)
Supplement: Supplementary file 3 — Appendix S1 [file CCR3-10-e6502-s001.docx]

**Video clips**

Video clip 1. 3D echocardiography for calculation of left ventricular ejection fraction (53%).

Video clip 2. Speckle-tracking echocardiography for calculation of left ventricular global longitudinal strain (-13.4%).
